# Ultra slow acoustic energy transport in dense fish aggregates

**DOI:** 10.1038/s41598-021-97062-4

**Published:** 2021-09-02

**Authors:** Benoit Tallon, Philippe Roux, Guillaume Matte, Jean Guillard, John H. Page, Sergey E. Skipetrov

**Affiliations:** 1 CNRS, ISTerre, University Grenoble Alpes, 38000 Grenoble, France; 2 iXblue, Sonar Division, 13600 La Ciotat, France; 3 CARRTEL, INRA, University Savoie Mont Blanc, 74200 Thonon-les-Bains, France; 4 Department of Physics and Astronomy, University of Manitoba, Winnipeg, MB R3T 2N2 Canada; 5 CNRS, LPMMC, University Grenoble Alpes, 38000 Grenoble, France

**Keywords:** Acoustics, Biological physics, Structure of solids and liquids

## Abstract

A dramatic slowing down of acoustic wave transport in dense fish shoals is observed in open-sea fish cages. By employing a multi-beam ultrasonic antenna, we observe the coherent backscattering phenomenon. We extract key parameters of wave transport such as the transport mean free path and the energy transport velocity of diffusive waves from diffusion theory fits to the experimental data. The energy transport velocity is found to be about 10 times smaller than the speed of sound in water, a value that is exceptionally low compared with most observations in acoustics. By studying different models of the fish body, we explain the basic mechanism responsible for the observed very slow transport of ultrasonic waves in dense fish shoals. Our results show that, while the fish swim bladder plays an important role in wave scattering, other organs have to be considered to explain ultra-low energy transport velocities.

## Introduction

Because of their swim bladder (analogous to an immersed air bubble), bony fish (*Osteichthyes*) are very strong scatterers for underwater acoustic waves. Thus, (multi-beam) sonar techniques are very efficient to locate and characterize shoals, aggregates or even isolated fish. Most of the fisheries acoustics methods are developed under the single scattering approximation^1^, i.e. for low fish concentrations when the wave is scattered at most once during propagation. For such low densities ($\sim 1$ fish/m$^3$), fish counting is straightforward and efficient using traditional methods such as echo-counting or echo-integration^2^. However, fish aggregates can be very dense ($\sim 50$ fish/m$^3$) for aquaculture purposes, for example, and in naturally occurring fish schools. In these cases, the backscattered signal received by the antenna is scattered by several fish during the wave’s propagation, which makes fish counting much more challenging. This multiple scattering regime is problematic for the aquaculture industry, for which nonintrusive biomass estimation is one of the most important issues in regular practice^3^. In a previous work^4^, we suggested the use of mesoscopic physics and, in particular, of multiple scattering theory based on the diffusion approximation to deal with wave propagation in dense fish shoals.

In the diffusion approximation, one assumes that after a propagation distance corresponding to the transport mean free path $\ell ^*$, the average intensity of multiply scattered waves follows a diffusion process (such as in heat diffusion) with a characteristic diffusivity $D = v_e \ell ^*/3$. The diffusivity involves the energy transport velocity of diffusive waves^5–7^ $v_e = 3D/\ell ^*$. The energy velocity is thus a key parameter describing wave transport, and is proportional to the ratio of energy flux to energy density. It is important to recall that this energy velocity $v_e$ is a quite different quantity to the more commonly encountered sound speeds, which are the phase and group velocities, $v_{ph}$ and $v_{gr}$, and which characterize the ballistic (“line-of-sight”) propagation of the configurationally averaged wave field (configurational averaging is only necessary in an inhomogeneous medium). By contrast, the diffusive energy velocity can be thought of as the local velocity of energy transport between scattering events in the multiply scattered wave “coda”, which for a pulsed experiment extends over a range of times (which can be very long) after the ballistic pulse has passed through the medium. For diluted or non-resonant systems, $v_e \simeq v_0$ (where $v_0$ is the sound speed in water). However, as demonstrated with previous model systems, the energy velocity can be highly impacted by resonant phenomena that are typically encountered when the acoustic wavelength becomes similar to the typical size of the scatterers^5–7^.

In this paper, we first report an observation of an ultra-low value of the energy transport velocity of diffusive acoustic waves in a dense fish shoal. Thus, rather than measuring the quite familiar phase velocity that has often been studied in the context of bubbly media^8^, we focus here on diffusive waves that are dominant in dense fish aggregates. The diffusive field and the corresponding intensity is probed through coherent backscattering (CBS) measurements^9–12^. CBS is a wave interference phenomenon that manifests itself by an enhancement (by a factor of 2) of the average backscattered intensity measured in the direction opposite to that of the incident wave. The angular profile of backscattered intensity has a cone shape in the stationary (continuous wave) limit, with a width that depends on $\ell ^*$. In the dynamic case, accessed using a pulsed source, the temporal evolution of the backscattering peak’s width depends on *D* and hence on $v_e$. In order to scan the angular dependence of the backscattered intensity, we employ a multi-beam sonar probe (based on the Seapix technological brick^13^) in a large fish cage anchored in open sea. In the second part of the paper, we present a comparative study of energy transport velocity calculations based on Mie theory^14,15^. The comparison of four scattering models for individual fish reveals that, while fish are usually approximated as air bubbles in water for scattering of acoustic waves, their complex structure can play an important role for wave transport in dense shoals. This comparison enables us to identify the essential features that need to be accounted for, and to propose a simple model to replicate the scattering properties of fish in dense shoals.

## Results

### Experiments

Coherent backscattering (or weak localization) is a mesoscopic phenomenon that has been observed for light^9,10^, ultrasound^11^, matter waves^16^ and seismic waves^17^. This effect is due to the constructive interference of waves following time-reversed pairs of paths. This interference produces a peak, centred on the exact backscattering direction, in the angular profile of backscattered intensity. In order to observe CBS, we use a Mills Cross multi-beam antenna made of two perpendicular ultrasonic arrays (2$\times$64 transducers with a central wavelength $\lambda =$ 1 cm). Experiments involve sending a short acoustic pulse (central frequency 150 kHz) into the fish cage by firing all transducers at the same time. We then perform an angular scan of the backscattered intensity using the beamforming method^18^ that overcomes some limitations encountered in the “classical” approach^11^ for which signals emitted and received with each transducer element are separately measured and analysed. Advantages of this beam-forming method include better angular resolution of the backscattering cone, improved signal to noise, and the ability to accurately measure *D* in dynamic measurements even when the array is not in the far field of the sample^18^. Hence it is the preferred method for accurately measuring the CBS profiles in experiments such as the ones reported in this paper. The transducer array is placed just below the surface of the water, facing the sea bottom and a shoal of gilthead sea breams (*Sparus aurata*). The average mass of the sea breams is 150 g and their concentration in the open cage is $\eta \sim 50$ fish/m$^3$.

**Figure 1:**
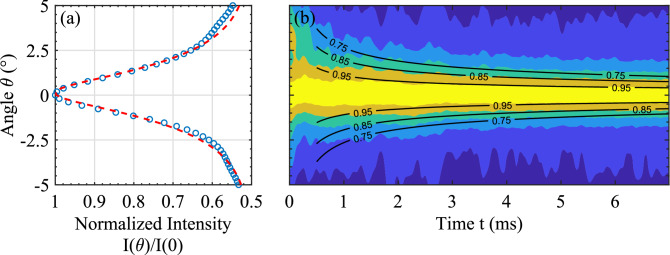
(**a**) Stationary CBS profile (blue open circles) fitted with the diffusion theory (red dashed line). (**b**) Dynamic CBS profile fitted with the diffusion theory (black solid lines). In (**b**), the intensity is normalized by its peak value at each time to show more clearly the temporal evolution of the width.

Time integration of the backscattered intensity yields the *stationary* CBS profile, which has an angular width of $\Delta \theta \sim \lambda /\ell ^*$ (where $\lambda$ is the wavelength in water). We fit the entire profile with the predictions of diffusion theory^12^; see Fig. 1a. The fitting parameters are the transport mean free path $\ell ^* =(6.0 \pm 0.2)$ cm and the absorption length $\ell _a =(100 \pm 3)$ cm, which characterizes the exponential decay of acoustic energy due to losses. The value $k\ell ^*= 36\gg 1$ (with $k = 2\pi /\lambda$) indicates that even though sound is strongly scattered in this shoal, no complex phenomena such as strong localization (occurring for $k\ell ^*\sim 1$) impact the CBS peak shape.

The time-resolved *dynamic* CBS peak narrows with time (Fig. 1b). From diffusion theory^12^, the CBS width depends on diffusivity as $\Delta \theta \propto 1/\sqrt{Dt}$. Using the measured value of $\ell ^*$ and the diffusion theory, the fitting of the dynamic CBS peak gives us $D = (1.3\pm 0.1)$ m$^2$/s. In this way, the simultaneous measurement of $\ell ^*$ and *D* leads to the energy velocity $v_e = 3D/\ell ^* = (65\pm 5)$ m/s. This value of $v_e$ is surprising low (an order of magnitude lower than the sound speed in water $v_0 =$ 1500 m/s) , which is a very rare observation for acoustic waves^4,6,7,19,20^. Similarly slow diffusion has been observed previously with CBS measurements in dense shoals of sea breams, sea basses (*Dicentrarchus labrax*) or croakers (*Argyrosomus regius*)^4^, but no such extreme behaviour has been found for acoustic waves in other multiply scattering media^6,7,19,20^. However, unlike this past work on dense fish shoals, the cage considered in this paper has a sufficiently low fish concentration to allow us to neglect mesoscopic interferences that might impact the energy velocity, thereby facilitating a quantitative interpretation of the current results. In the following section, we employ a model^15^ that takes into account the scattering delay induced by the fish and explains this ultra low value of energy velocity.

### Energy velocity calculation

Here, we employ a microscopic model of the energy velocity^15^ in order to identify the mechanisms responsible for the ultra low $v_e$ value. This description is based on a model that accounts for the delay $\Delta t_{ave}$ induced by an immersed scatterer, and predicts a result for $v_e$ that may be approximated as^20^

1 $$\begin{aligned} \frac{1}{v_e} \approx \frac{1}{v_{gr}} + \eta \sigma _T\Delta t_{ave}. \end{aligned}$$

Here $v_{gr}$ is the group velocity of the average wave field ($v_{gr} \sim v_0$ in the absence of dispersion effects) and $\eta$ is the scatterer concentration. $\Delta t_{ave}$ is the scattering delay time for a single scattering event, calculated from the intensity-weighted angle-averaged phase derivative with frequency of the scattering amplitude [Eq. (4)], and $\sigma _T$ is the total scattering cross section. Thus, to obtain slow diffusive waves, the system needs to contain a sufficiently high concentration (high $\eta$) of strong scatterers (high $\sigma _T$) capable of inducing a large scattering delay (large $\Delta t_{ave}$).

**Figure 2:**
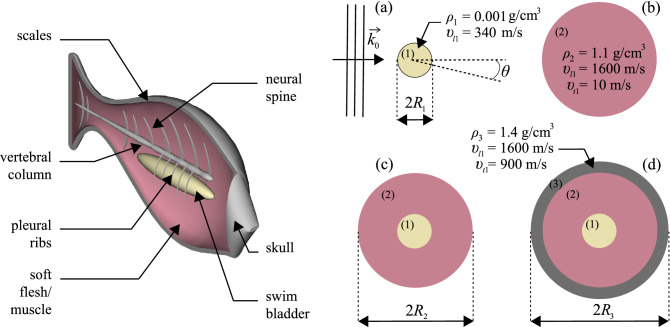
On the left: schematic representation of a sea bream body. On the right: models (**a**)–(**d**) used for the energy velocity calculation.

In order to identify the parts of the fish that are responsible for the ultra-low energy velocity, we use Eq. (1) to compute $v_e$ for four idealized spherically symmetric scattering models representing different simplifications of the complex fish body (Fig. 2). Since this theory enables analytic expressions to be obtained for relatively simple scattering geometries, the goal here is to capitalise on this capability to search for the key features that need to be considered in order to understand the origin of the remarkably slow energy velocity. Thus, rather than attempting a complex simulation that might obscure the basic scientific mechanism(s) at play, we focus on very simple models to reveal the basic scattering mechanisms involved:

- *Model a* an air bubble representing the fish swim bladder with radius $R_1 =$ 10 mm (Fig. 2a), longitudinal wave speed $v_{l1} = 340$ m/s and density $\rho _1 =$ 0.001 g/cm$^3$.
- *Model b* a homogeneous soft sphere representing the fish flesh with radius $R_1 =$ 76 mm, longitudinal wave speed $v_{l2} = 1600$ m/s, shear wave speed $v_{t2} = 10$ m/s and density $\rho _2 =$ 1.1 g/cm$^3$ (Fig. 2b).
- *Model c* a combination of models a and b representing the swim bladder surrounded by a flesh layer (Fig. 2c).
- *Model d* similar to model c with an additional hard thin layer representing fish scales and bones with radius $R_3 =$ 78 mm, longitudinal wave speed $v_{l3} = 1600$ m/s, shear wave speed $v_{t3} = 900$ m/s and density $\rho _3 =$ 1.4 g/cm$^3$ (Fig. 2d).

Some scattering theories allow calculations for spheroids^21^ that might be closer to the actual fish shape, but none of those calculations are developed for multilayer scatterers. However, the spherical approximation is suitable in the present case because of the randomized fish orientation in the azimuthal plane. Thus on average, the effective scatterer shape seen by the incident plane wave can be approximated as a sphere.

**Figure 3:**
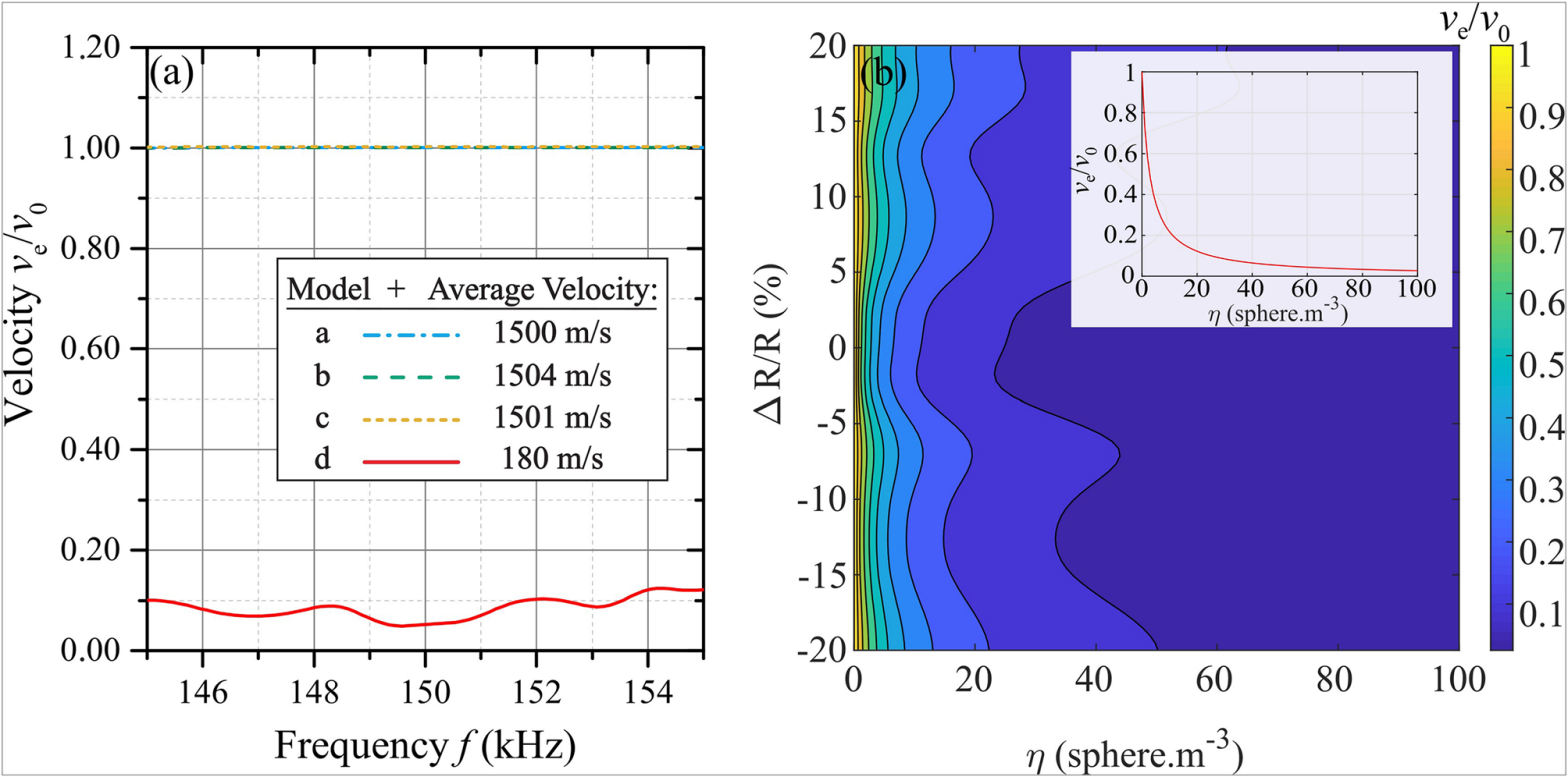
(**a**) Normalized velocity $v_e/v_0$ calculations versus frequency for the four different models (see the legend for the average value over this frequency range of the energy velocity for each model). (**b**) Normalized velocity $v_e/v_0$ calculations versus fish size variation $\Delta R/R$ and concentration $\eta$ at a given frequency $f =$ 150 kHz for model d. The inset represents $v_e/v_0$ versus fish concentration for $\Delta R/R$ = 0 and $f =$ 150 kHz.

Model a represents the usual assumption in fisheries acoustics^1,22^: since, at least near resonance, the swimbladder is the most reflective organ for acoustic waves, fish shoals are often seen as clouds of air bubbles in water. However, as shown in Fig. 3a, Eq. (1) applied to model a fails to explain the ultra-low value of energy velocity found in our experiments. The same conclusion can be drawn for models b and c, for which we also obtain $v_e\sim v_0$. On the other hand, model d predicts a very slow energy velocity, $v_e \approx$ 100 m/s, at the frequency $f =$ 150 kHz, and ranges from about 50–150 m/s over the bandwidth of the transducers. In comparison with the other models, these values are very close to our measurement ($v_e = 65\pm 5$ m/s). The phase $v_{ph}$ and group velocities $v_{gr}$ (see Supplementary Information, Fig. S2) can also be calculated for these models. Both of these velocities are found to be very close to $v_0$ for *all* four models in this frequency range. These results indicate that the complex fish structure mostly impacts only the diffusive waves; the ballistic wave velocities (the “line-of-sight” propagation through the shoal) are not significantly affected by the fish scattering and concentration. Thus, even though it would be relatively simple to measure $v_{ph}$ and $v_{gr}$ using well-established pulse-echo or transmission techniques (see Ref.^23^ for a relatively recent example of the former case), such measurements would not be expected to be very useful for fish monitoring in aquaculture environments, at least under the current experimental conditions.

Thus, the hard thin layer surrounding the soft solid representing fish flesh seems to play an important role for the slowing down of diffusive acoustic waves. Figure 3b shows the calculation of energy velocity for a range of fish concentrations $\eta$ and as a function of size variation $\Delta R/R$ (here the size variation $\Delta R/R$ is a factor that is equally applied to the three radii $R_1$, $R_2$ and $R_3$). It is important to note the weak size dependence of energy velocity on $\Delta R/R$ that proves that the drop of $v_e/v_0$ in model d with respect to model c is not due to size differences (outer radius $R_3$ versus $R_2$). In contrast, the fish concentration $\eta$ seems to have a significant impact on $v_e$. This effect could be interesting for enabling a new method of biomass assessment with acoustic waves compared with more traditional acoustic methods (see, for example, Ref.^2^).

## Discussion

In this section, we interpret the role of each part of the fish model on the scattering delay. The double core-shell structure of model d seems to explain the observed ultra-low energy velocity. As has been observed in the past^24^, core-shell scatterers can indeed exhibit very strong scattering. However, no slow diffusive waves have been measured for such scatterers in the past. Resonant mechanisms have been identified^5–7^ as being responsible for the decrease of $v_e$ in both optics and acoustics, but only for homogeneous scatterers. These effects result in large energy velocity variations, with $v_e \sim v_0$ far from resonant frequencies and $v_e \ll v_0$ around resonances. In the present case, such frequency variations (expected over several tens of kHz) cannot be observed in our experiments, since the sonar bandwidth is too narrow; hence, frequency-resolved measurements were not feasible, and our experimental determination of $v_e$ corresponds to a narrow bandwidth-limited average around the central frequency (150 kHz) of the transducers. Thus, from experimental observations, we can measure conclusively the low $v_e$ value but we cannot conclude anything about its potential variations over a larger frequency range.

**Figure 4:**
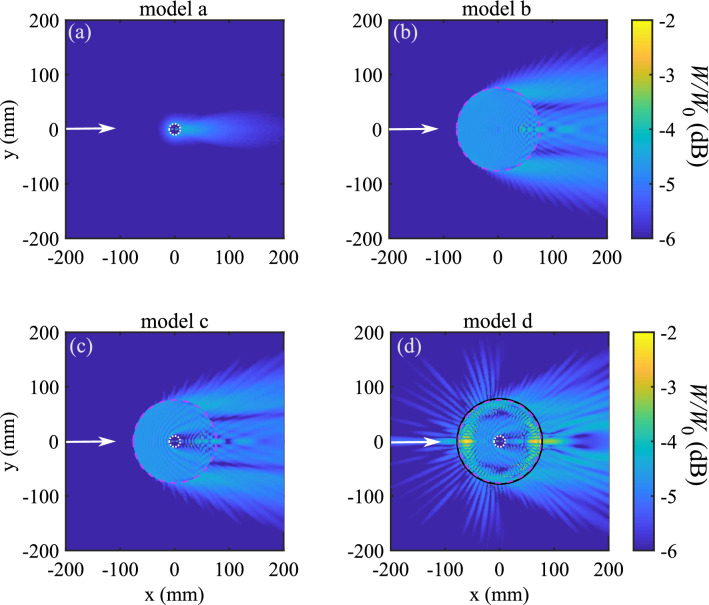
Calculations of the longitudinal acoustic energy density *W* for the four different model scatterers at $f=150$ kHz. $W_0$ is the incident energy density. The white dotted line represents the limits of the swim bladder, the dashed pink line the flesh layer and the solid black line the thin scales and bones layer. White arrows indicates the direction of the incident plane wave.

A way to interpret the scattering delay impacting the energy velocity is the calculation of the acoustic energy density inside and outside the scatterer^20,25^. High values of energy density suggest that waves are “stored” in the scatterer. This energy is then released into the surrounding medium with a certain delay resulting in a slowing down of diffusive wave transport. Figure 4 shows the energy density calculations for longitudinal waves at the frequency $f =$ 150 kHz for the four different model scatterers.

The energy density calculation for an air bubble (model a) exhibits predominantly forward scattering as expected at high frequency for small scatterers with large acoustic contrast^26^. Since the frequency being considered in this study is far from the resonance of the bubble^8^ ($f_{res} \sim$ 0.5 kHz), the scattering is very weak for this model, and the energy density inside the scatterer is small. Thus, one might expect only a modest scattering delay, and the scattering strength (or total cross section $\sigma _T$) induced by the isolated swim bladder is not high enough for the product of scattering delay and cross section to cause a significant decrease of $v_e$. For models b and c, both the energy density inside the scatterers and the forward scattering are somewhat larger than for model a, suggesting that there could be a bigger difference between group and energy velocities, but additional information would be needed to assess if they could be interesting candidates for predicting a slow energy velocity. However, for the double core-shell system (model d), the result of the energy density calculation is much more striking, as we observe a *very* strong increase of scattered energy density (the large increase in wave energy stored in the fish body is due to the hard scales and bones layer). The subwavelength-scale outer layer helps the generation of slow shear waves via mode conversion and the trapping of both longitudinal and shear waves in the fish flesh. This stored acoustic energy is then re-radiated into the surrounding water with a large delay^15^.

**Figure 5:**
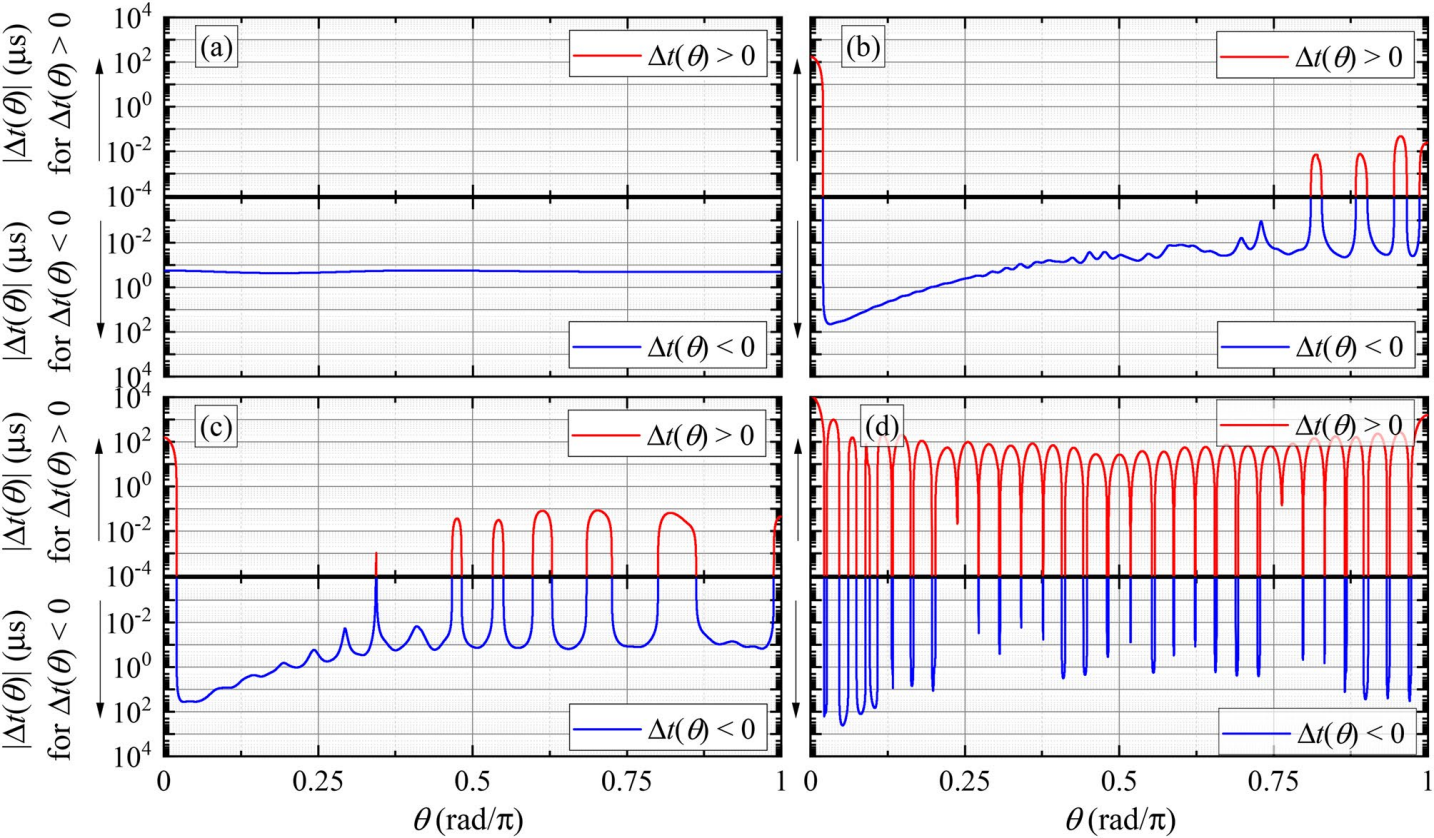
(**a**)–(**d**) Angular dependence of the normalized scattering delays $\Delta t_{\theta }$ calculated for the four models (a, b, c and d), at a frequency of 150 kHz.

The link between the stored energy and the large scattering delay becomes clear when comparing Figs. 4 and 5, which shows the angular dependence of the scattering delay $\Delta t(\theta )$ for all models (Fig. 5a–d) (see the Supplementary Information for calculation details). In particular, this figure shows that the angle-resolved scattering delays are much larger, typically by a couple of orders of magnitude, for model d than for the other models, for which the energy densities inside the scatterers are much less. Specifically, for model a, the delay is relatively small and negative at all scattering angles, whereas for models b and c, the delays are negative for most angles and have, typically, somewhat larger magnitudes. In all three cases, these results suggest that the total angle averaged delay will be fairly small and certainly negative (indicating a slight enhancement of the energy velocity relative to the group velocity). For model d, however, very large delays, both positive and negative, are seen, with the positive delays dominating. These observations are confirmed by doing the angular integration of $\Delta t(\theta )$, yielding average scattering delays per wave period $\Delta t_{\text {ave}}/T_0$ for models a through d of $-0.4, -1.4, -1.4$ and 80.8, respectively. Thus, we find that adding the hard coating increases the magnitude of the angle-averaged delay by a factor of approximately 100 or more in comparison with the other models.

**Figure 6:**
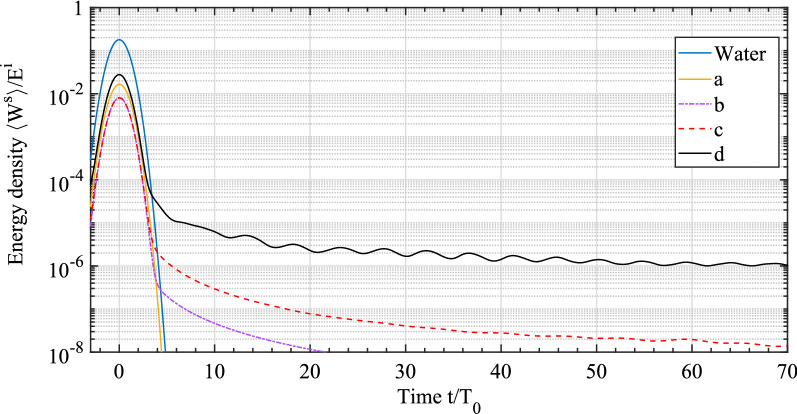
Temporal evolution of scattered energy density $\langle W^s \rangle$ (plots are normalized by the total incident energy density $E^i$). The solid blue line represents the energy density of incident pulse (without a scatterer). The time axis is normalized by the width of the incident pulse $T_0 =$ 0.1 ms.

To further illustrate this large scattering delay, we calculate the dynamic energy density of the scattered waves $\langle W^s\rangle$ outside the scatterers (Fig. 6). $\langle W^s\rangle$ is obtained by integrating *W*(*f*) (c.f., Fig. 4 at $f=150$ kHz) over the bandwidth of the incident pulse. The resulting temporal evolution of *W* is spatially averaged on the region $R_3<r<b$ (where $r = \sqrt{x^2 + y^2}$ and $b =$ 85 mm is the average inter-scatterer distance for the concentration $\eta$ considered here. In this way, we obtain the dynamics of the acoustic energy density in the surroundings of each scatterer.

The higher overall energy obtained with model d confirms the strong influence on the scattering induced by the presence of scales and bones. Furthermore, while models a, b and c all predict that $\langle W_d \rangle$ decreases quickly (which explains $v_e\sim v_0$ for these systems), a much slower decay of $\langle W_d \rangle$ with time is obtained with model d. This slow decay for model d demonstrates clearly that the large stored acoustic energy in the fish body (Fig. 4d) is slowly radiated into the surrounding medium (Fig. 6). To summarize, the core-shell model system with swim bladder + flesh + scales-and-bones layer leads to a large slowly decaying energy density associated with a large scattering delay. These observations and the quite large fish concentration $\eta$ successfully explain the slow diffusion of acoustic waves observed in the sea bream shoal.

## Conclusions

While the assumption that fish shoals are equivalent to air bubble clouds is adequate for many acoustic studies^1^, some cases with dense fish shoals have to be considered more carefully. Indeed, high fish concentration ($\eta \sim$ 50–100 fish/m$^3$) can lead to an accumulation of scattered energy, a resulting increase in scattering delay and a drastic slowing down of sound diffusion. Thus, it is essential to properly estimate $v_e$ in fish shoals in order to extend the range of application of acoustic fisheries techniques to very dense shoals. Several improvements (such as scatterer shape and structure) can, in principle, be implemented in the model in order to calculate more accurate $v_e$ values. Nonetheless, as a first study, the simple models used here are sufficient to reveal the essential wave physics behind our observations, and enable us to demonstrate the strong impact of coating layers surrounding the swim bladder on wave transport in dense shoals.

As shown in Fig. 3b, the strong energy velocity dependence on the fish concentration could be useful for fish counting purposes. The method is particularly interesting since $v_e$ depends strongly on concentration and only weakly on fish size variation (over a reasonable size range for aquaculture conditions: $\Delta R/R\leqslant \pm$ 15%). In particular, such a tool could help the aquaculture industry for which large fish concentrations make acoustic biomass estimation impossible using traditional acoustic approaches based on the assumption of single scattering. Noninvasive monitoring of fish farms using diffusive waves^4,27^ is currently under study with long-term experiments (several weeks) in order to investigate the impact of fish size and biomass variations on the diffusive transport of sound waves.

## Methods

Experiments have been conducted with the approval of ethics committee/IRB of *Cannes Aquafrais* and in accordance with ARRIVE guidelines. During experiments, no fish have been manipulated and the usual working routine of the fish farmers have not been modified. The experiments were carried out according to the experimental protocols used within the framework of the studies on fish by a method recognized as not intrusive and with the scientific and technical validation of the institute guarantor of the project (CNRS, European Maritime and Fisheries Fund, Osiris program PFEA470017FA1000007). The normal behaviour of the fish is not affected by the experiments, which are conducted under regulations imposed by a breeding authorization from the Ministry of the Environment.

The sea cages are located in the Mediterranean Sea (Cannes, France) where the water temperature is about $20^{\circ }$ and salinity 3.6%. The cage in which experiments were conducted is cubic with a volume of 125 m$^3$. The distance from the bottom of the cage to the sea bottom is $z =$ 6.5 m. To maintain the organic label of the farm and to avoid the need for drug treatments, the fish densities in these cages are lower than those in intensive farming facilities (where mass densities can reach 100 kg/m$^3$). The feeding procedures are controlled to obtain a calibrated fish size.

The theory used to fit the experimental CBS data is derived from a diffusion equation for the average intensity $$\langle I({\mathbf {r}},t) \rangle$$^12^ that is solved for a disordered medium occupying the half-space $$z > 0$$, with a delta-function source at $$z = z'=\ell ^*$$^28^:

2 $$\begin{aligned} \langle I(t) \rangle = \frac{I_0}{2\pi }\int _{-\infty }^{+\infty }\frac{z_0{\text {exp}}(-\gamma _0 z')}{D(1+\gamma _0 z_0)}{\text {exp}}(-{\text {i}}\Omega t)d\Omega , \end{aligned}$$

where $\gamma _0^2(\Omega ) = \frac{-{\text {i}}\Omega }{D} + \frac{1}{D\tau _a}$, $\tau _a$ is the characteristic absorption time and $z_0 = \frac{2}{3}\frac{1+R}{1-R}\ell ^*$ is the extrapolation length, with $R =$ 0.99 as the reflection coefficient for the water/air interface. Theoretical predictions for both the dynamic and stationary CBS peaks can then be obtained using this theory^4^.

The energy velocity theory^5^ (Eq. 1) is based on the calculation of the scattering function $F(\theta ) = |F(\theta )|{\text {e}}^{{\text {i}}\varphi (\theta )}$ with magnitude $|F(\theta )|$ and phase $\varphi (\theta )$. $F(\theta )$ represents the scattering amplitude in the direction given by the angle $\theta$ with respect to the incident wavevector, and is given by the following expression:

3 $$\begin{aligned} F(\theta ) = \frac{1}{ik_{\text {0}}}\sum _{\text {n}}(2{\text {n}} + 1)A_{\text {n}}P_{\text {n}}(\cos \theta ), \end{aligned}$$

where $k_0$ represents the wavenumber of incident wave, $P_n$ the Legendre polynomials and $A_n$ the scattering amplitude coefficients of the scattered field. The $A_n$ coefficients are obtained by solving the Mie problem^14^, invoking the calculation of stress and displacement continuity conditions (for both longitudinal and shear waves) at the boundaries $R_1$, $R_2$ and $R_3$^29^. $F(\theta )$ is then used to obtain the scattering cross-section $\sigma$ and delay $\Delta t_{ave}$ that are needed for the calculation of Eq. (1):

4 $$\begin{aligned} \sigma = 2\pi \int {d\theta \sin \theta \left| f(\theta ) \right| ^2}\ \quad {\text {and}}\quad \Delta t_{ave} = \frac{\int {d\theta \sin \theta \left| f( \theta ) \right| ^2}\frac{\partial \varphi }{\partial \omega } }{\int {d\theta \sin \theta \left| f( \theta ) \right| ^2}}. \end{aligned}$$

Additional details on the experimental method and theoretical calculations can be found in the Supplementary Information.

## Supplementary Information

Supplementary Information.
