# Supplementary material for: Ultra slow acoustic energy transport in dense fish aggregates
Source: Sci Rep. 2021 Sep 2;11:17541. doi: 10.1038/s41598-021-97062-4 (PMC8413328; doi:10.1038/s41598-021-97062-4)
Supplement: Supplementary file 1 — Supplementary Information. [file 41598_2021_97062_MOESM1_ESM.pdf]

Supplementary Information for "Ultra-Slow Acoustic Energy Transport in Dense Fish Aggregates"

Benoit Tallon,[1] Philippe Roux,[1, a)] Guillaume Matte,[2] Jean Guillard,[3] John H. Page,[4] and Sergey E. Skipetrov[5]
[1)] *Univ. Grenoble Alpes, CNRS, ISTerre, 38000 Grenoble, France*
[2)] *iXblue, Sonar division, 13600 la Ciotat, France*
[3)] *Univ. Savoie Mont Blanc, INRA, CARRTEL, 74200 Thonon-les-Bains, France*
[4)] *University of Manitoba, Department of Physics & Astronomy, Winnipeg, Manitoba R3T 2N2, Canada*
[5)] *Univ. Grenoble Alpes, CNRS, LPMMC, 38000 Grenoble, France*

(Dated: 2 March 2021)

[a)] To whom correspondence should be addressed; E-mail: philippe.roux@univ-grenoble-alpes.fr

INTRODUCTION

This document provides additional information on the experiments that were performed to investigate dense fish shoals in open-sea fish cages, and on the theory used to interpret the results of these measurements.

EXPERIMENTAL SET-UP

The CBS experiments were performed using a Mills cross-shaped antenna that consists of two perpendicular linear arrays, each with 64 ultrasonic piezoelectric transducers. Each transducer is narrow band (bandwidth, 10 kHz) with a central frequency of 150 kHz, which corresponds to a wavelength $\lambda \simeq$ of 1 cm in water. The transducers are square in shape and have sides of 0.5 cm $\simeq \lambda/2$. The antenna (expressly designed by the iXblue company for aquaculture monitoring) is placed just below the water surface, facing the sea bottom.

At full power, the radiation pressure of the ultrasonic antenna is 133 dB$\cdot\mu$Pa (measured at a distance $d = 1$ m from the source and for the frequency $f = 150$ kHz). The hearing threshold for sciaenidae fish is about 60 dB$\cdot\mu$Pa in the frequency range $f \in [0.1-1]$ kHz[S1]. No avoidance behavior of the fish has been observed during our experiments. For comparison, some vessel noise signatures are reported in Ref. S2 and turn out to be around 130 dB$\cdot\mu$Pa in the hearing frequency range of sciaenidae fish.

DIFFUSION THEORY

As indicated in the Methods section of the main paper, the theory used to interpret the experimental CBS data is obtained from a diffusion model for the average intensity[S3] $\langle I(\mathbf{r},t)\rangle$ in a disordered medium occupying the half-space $z > 0$ when the source is a delta-function at $z = z' = \ell^{*}$[S4]. The expression for $\langle I(\mathbf{r},t)\rangle$ is repeated here for convenience:

$$\langle I(t)\rangle = \frac{I_0}{2\pi}\int_{-\infty}^{+\infty} \frac{z_0 \exp(-\gamma_0 z')}{D(1+\gamma_0 z_0)} \exp(-\mathrm{i}\Omega t)\, d\Omega, \tag{S1}$$

where $\gamma_0^2(\Omega) = \frac{-\mathrm{i}\Omega}{D} + \frac{1}{D\tau_a}$, τ_a is the characteristic absorption time and $z_0 = \frac{2}{3}\frac{1+R}{1-R}\ell^{*}$ is the extrapolation length, with $R = 0.99$ as the reflection coefficient for the water/air interface. The theoretical expression used to fit the dynamic CBS profile $\langle I(\theta,t)\rangle$ follows from the same diffusion theory, and is given by

$$\langle I(\theta,t)\rangle = \frac{I_0}{2\pi}\int_{-\infty}^{+\infty} \frac{z_0}{D}\left\{ \frac{\mathrm{e}^{-\gamma_0 z'}}{1+\gamma_0 z_0} + \frac{\mathrm{e}^{-\gamma z'}}{1+\gamma z_0} \right\} \exp(-\mathrm{i}\Omega t)\, d\Omega, \tag{S2}$$

where $\gamma^2(\theta,\Omega) = \frac{-\mathrm{i}\Omega}{D} + k_0^2 \sin^2(\theta) + \frac{1}{D\tau_a}$ and $\gamma_0 = \gamma(\theta = 0,\Omega)$. The expression for the stationary CBS profile is obtained by integrating equation (S2) over time[S5].

PHASE AND GROUP VELOCITIES

We outline the calculation of the wave number k for the average field. This wave number, useful for the energy velocity calculation, is expressed as :

$$k^2 = k_0^2 + 4\pi\eta F(\theta = 0), \tag{S3}$$

where η is the scatterer concentration and $k_0 = \omega/v_0$ is the wave number of the incident wave at frequency $f = \omega/2\pi$ in water. In the far field approximation, the scattering function $F(\theta) = |F(\theta)|\exp[i\varphi(\theta)]$, with magnitude $|F(\theta)|$ and phase $\varphi(\theta)$, represents the scattering amplitude in the direction given by the angle θ with respect to the incident wave vector. $F(\theta)$ is given by the following expression

$$F(\theta) = \frac{1}{ik_0}\sum_{\mathrm{n}}(2\mathrm{n}+1)A_{\mathrm{n}}P_{\mathrm{n}}(\cos\theta), \tag{S4}$$

where P_n represents the Legendre polynomials and A_n the scattering amplitude coefficients of the scattered field. The A_n coefficients are obtained by solving the Mie problem[S6]: by imposing stress and displacement continuity conditions at the interfaces of a spherical scatterer (model a), one obtains a system of 3 linear equations with 3 coefficients[S7]. The solution of this system of equations requires inverting a 3×3 matrix, and leads to all of the 3 partial wave amplitudes (the scattered longitudinal wave, the refracted longitudinal wave and the refracted shear wave). The calculation of scattering amplitude of a coated sphere (model c) is obtained by solving a system of 7 linear equations with 7 coefficients, as detailed in Ref. S8. Following the same procedure, the last case of a double coated sphere (model d) requires the solution of a system of 10 linear equations with 10 coefficients given by stress and displacement continuity conditions at the 3 interfaces.

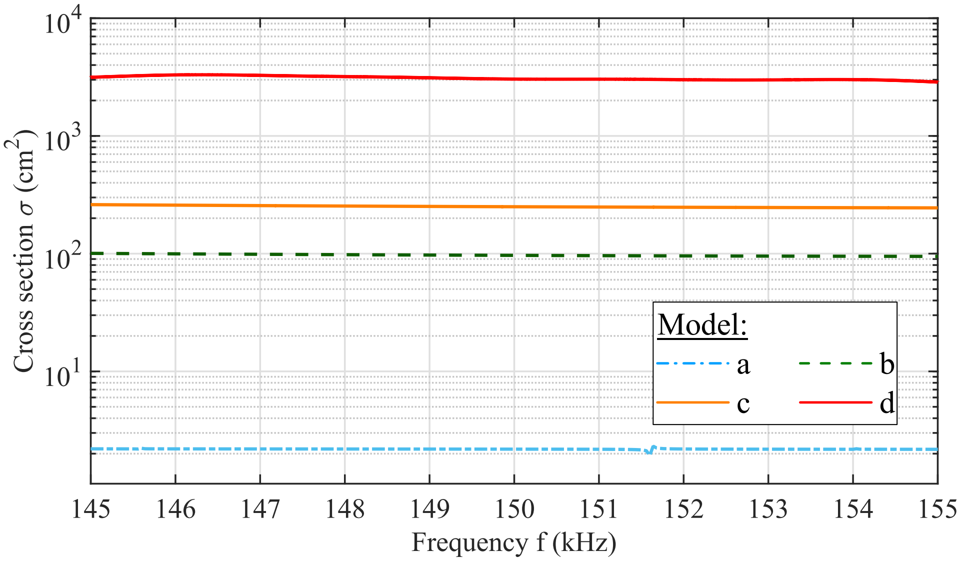

FIG. S1: Scattering cross section calculated for the four model scatterers.

The scattering function allows the calculation of the scattering cross-section σ to be performed:

$$\sigma = 2\pi\int d\theta\sin\theta\,|F(\theta)|^2. \tag{S5}$$

The scattering cross-section of our four spherical scatterers (models a-d) is represented in figure S1. As expected from the energy density calculations (Fig. 4), the scattering from the simple air bubble is weak in comparison with the more elaborate spherical models b, c and d. The significantly larger scattering strengths for the three more complicated spherical models indicate that they are more promising candidates for achieving slower wave transport, especially model d. However

it is evident that scattering strength alone is not sufficient to explain the energy velocity predictions obtained from these models (see Fig. 3(a) of the main paper).

The knowledge of the wave number k leads to the calculation of the phase velocity $v_p = \omega/\mathrm{Re}[k]$ and the group velocity $v_{\mathrm{gr}} = \partial\omega/\partial\mathrm{Re}[k]$. Both these velocities are plotted in figure S2 for the four model scatterers. In all cases, both of these velocities are very close to the water velocity v_0. These calculations reveal the weak phase shift of the forward scattered waves (since v_p and v_{gr} only depend on $F(\theta = 0)$ and $\partial\mathrm{Re}[F(\theta = 0)]/\partial\omega$, respectively). In terms of energy transport, these results indicate that the transport velocity of the average wave field (v_{gr}) is barely affected by the scattering. Thus, the ultra-slow transport of diffusive waves must result only from the very large scattering delay of multiply scattered waves that is described in the next section.

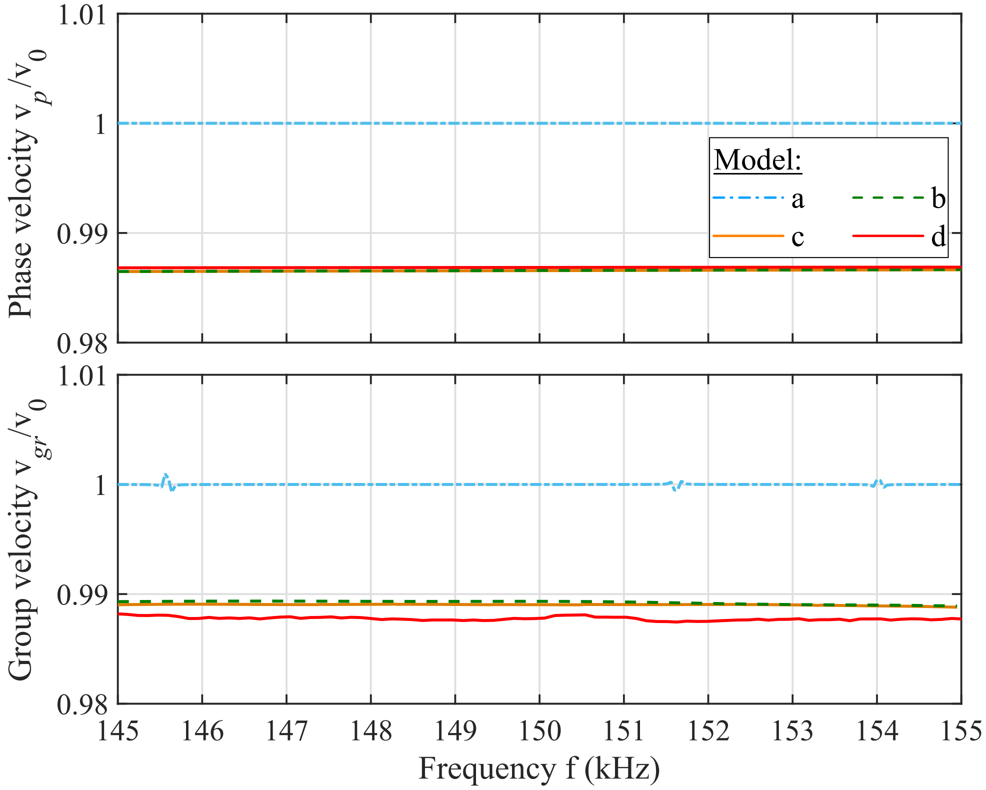

FIG. S2: Phase (upper panel) and group (lower panel) velocities calculated for the four different models.

ENERGY VELOCITY

The energy velocity calculation is based on the renormalization of the sound speed in pure water due to the scattering delay[S10,S11]

$$v_{\mathrm{e}} = \frac{v_0^2/v_{\mathrm{p}}}{1+\delta}, \tag{S6}$$

where the delay parameter δ is equal to

$$\delta = 2\pi\eta v_{\mathrm{gr}} \left(\frac{v_{\mathrm{p}}}{\omega} \frac{\partial \mathrm{Re} F(0)}{\partial\omega} + \int_0^\pi \sin(\theta) |F(\theta)|^2 \frac{\partial\varphi(\theta)}{\partial\omega} \mathrm{d}\theta \right). \tag{S7}$$

Under the approximation $v_0^2/v_p v_{gr} \approx 1$ (for weak dispersion of the average wave field), the energy velocity can be rewritten as[S12]

$$\frac{1}{v_e} = \frac{1}{v_{gr}} + \eta\sigma\Delta t_{ave}, \tag{S8}$$

where the average scattering delay Δt_{ave} is equal to

$$\Delta t_{ave} = \frac{\int d\theta \sin\theta \, |F(\theta)|^2 \frac{\partial\varphi(\theta)}{\partial\omega}}{\int d\theta \sin\theta \, |F(\theta)|^2} = 2\pi \int d\theta \sin\theta \Delta t(\theta). \tag{S9}$$

The right side of this equation follows from the definition of the total scattering cross section [equation (S5)], and is expressed in terms of the angle-dependent scattering delay $\Delta t(\theta) = |F(\theta)|^2 \frac{\partial\varphi(\theta)}{\partial\omega}/\sigma$. While the average-field velocities only involve the scattering function in the forward direction, the scattering delay of diffusive waves depends on the frequency derivative of phase shift in *all* scattering directions $\partial\varphi(\theta)/\partial\omega$. The group velocity being similar to the sound speed in pure water ($v_{gr} \sim v_0$), it is this scattering delay that is responsible for the remarkably slow values of the energy velocity obtained for the multi-layer sphere (model d). Thus, the adding of the hard scales layer significantly slows down the diffusive wave transport by facilitating the storing of acoustic energy within the scatterer, and the releasing of this energy with a large delay.

REFERENCES

[S1] F. Ladich and R. R. Fay, *Rev. Fish Biol. Fish.* **23**, 317 (2013).

[S2] J. Simmonds and D. MacLennan, *Fisheries Acoustics: Theory and Practice*, 2nd edition (Blackwell Science, Oxford, 2005).

[S3] E. Akkermans and G. Montambaux, *Mesoscopic Physics of Electrons and Photons* (Cambridge Univ. Press, 2007).

[S4] H.S. Carslaw and J.C. Jaeger, *Conduction of Heat in Solids* (Oxford science publications, 1959).

[S5] B. Tallon and P. Roux and G. Matte and J. Guillard and S. E. Skipetrov, *AIP Adv.* **10**, 055208 (2020).

[S6] G. Mie, *Ann. Phys.-Berlin* **25**, 377 (1908).

[S7] J. J. Faran, *J. Acoust. Soc. Am.* **23**, 405 (1951).

[S8] X. Jing and P. Sheng and M. Zhou, *Phys. Rev. A* **46**, 6513 (1992).

[S9] B. A. van Tiggelen and A. Lagendijk and M. P. van Albada and A. Tip, *Phys. Rev. B* **45**, 12233 (1992).

[S10] M. P. van Albada and B. A. van Tiggelen and A. Lagendijk and A. Tip, *Phys. Rev. Lett.* **66**, 3132 (1991).

[S11] B. Tallon and T. Brunet and J. Leng and J. H. Page, *Phys. Rev. B.* **101**, 054202 (2020).

[S12] H. P. Schriemer and M. L. Cowan and J. H. Page and P. Sheng and Z. Liu and D. A. Weitz, *Phys. Rev. Lett.* **79**, 3166 (1997).
